# Postprandial Responses to Lipid and Carbohydrate Ingestion in Repeated Subcutaneous Adipose Tissue Biopsies in Healthy Adults

**DOI:** 10.3390/nu7075224

**Published:** 2015-07-01

**Authors:** Aimee L. Dordevic, Felicity J. Pendergast, Han Morgan, Silas Villas-Boas, Marissa K. Caldow, Amy E. Larsen, Andrew J. Sinclair, David Cameron-Smith

**Affiliations:** 1 School of Exercise & Nutrition Sciences, Deakin University, Victoria 3125, Australia; E-Mails: fpenderg@deakin.edu.au (F.J.P.); marissa.caldow@unimelb.edu.au (M.K.C.); a.larsen@latrobe.edu.au (A.E.L.); d.cameron-smith@auckland.ac.nz (D.C.-S.); 2 Department of Nutrition & Dietetics, Monash University, Victoria 3168, Australia; E-Mail: andrew.sinclair@deakin.edu.au; 3 School of Biological Sciences, University of Auckland, Auckland 1142, New Zealand; E-Mails: t.han@auckland.ac.nz (H.M.); s.villas-boas@auckland.ac.nz (S.V.-B.); 4 Basic and Clinical Myology Laboratory, Department of Physiology, University of Melbourne, Victoria 3010, Australia; 5 Department of Physiology, Anatomy and Microbiology, College of Science, Health and Engineering, LaTrobe University, Victoria 3086, Australia; 6 Metabolic Research Unit, Deakin University, Waurn Ponds, Victoria 3216, Australia; 7 Liggins Institute, University of Auckland, Auckland 1142, New Zealand

**Keywords:** adipokine, adipose tissue, biopsy, inflammation, postprandial

## Abstract

Adipose tissue is a primary site of meta-inflammation. Diet composition influences adipose tissue metabolism and a single meal can drive an inflammatory response in postprandial period. This study aimed to examine the effect lipid and carbohydrate ingestion compared with a non-caloric placebo on adipose tissue response. Thirty-three healthy adults (age 24.5 ± 3.3 year (mean ± standard deviation (SD)); body mass index (BMI) 24.1 ± 3.2 kg/m^2^, were randomised into one of three parallel beverage groups; placebo (water), carbohydrate (maltodextrin) or lipid (dairy-cream). Subcutaneous, abdominal adipose tissue biopsies and serum samples were collected prior to (0 h), as well as 2 h and 4 h after consumption of the beverage. Adipose tissue gene expression levels of monocyte chemoattractant protein-1 (MCP-1), interleukin 6 (IL-6) and tumor necrosis factor-α (TNF-α) increased in all three groups, without an increase in circulating TNF-α. Serum leptin (0.6-fold, *p* = 0.03) and adipose tissue leptin gene expression levels (0.6-fold, *p* = 0.001) decreased in the hours following the placebo beverage, but not the nutrient beverages. Despite increased inflammatory cytokine gene expression in adipose tissue with all beverages, suggesting a confounding effect of the repeated biopsy method, differences in metabolic responses of adipose tissue and circulating adipokines to ingestion of lipid and carbohydrate beverages were observed.

## 1. Introduction

Dysregulated metabolism and inflammation in adipose tissue, a major metabolic tissue and destination of ingested nutrients [1,2], is a core feature of chronic metabolic diseases such as cardiovascular disease and Type 2 Diabetes. Upon entering adipocytes, macronutrients are likely to impact on the metabolic state of the adipose tissue, including modulating expression levels of genes and the synthesis of adipokines, which are implicated in whole body energy homeostatic and immune regulation [3,4,5,6].

Adipokine expression levels, in response to meals, are regulated by both the composition of the meal and the metabolic state of the individual. Ingestion of either fat or carbohydrate meals mitigate the decline of circulating leptin that occurs during fasting in both in healthy and obese humans [7,8]. Whereas increased interleukin-6 (IL-6) secretion from adipose tissue positively correlates with insulin-resistance in obese subjects [9]. One study demonstrated that adipose tissue adiponectin gene expression levels were reduced post-meal ingestion in type 2 diabetics, yet remain unaltered in non-diabetic weight-matched individuals [10]. Postprandial inflammatory responses in adipose tissue have also been demonstrated in individuals with metabolic syndrome regardless of background diet and meal composition [11], as well middle-aged adults, irrespective of fat type [12]. However, repeated biopsy procedures have been demonstrated to influence inflammatory responses in skeletal muscle [13], yet have not been investigated in adipose tissue or postprandial studies.

Aside from these studies addressing the impact of obesity and metabolic disease on the postprandial inflammatory response of adipose tissue, there is no clinical data examining the usual response of adipose tissue to differing macronutrients in reference to a healthy population compared to a non-caloric placebo. This study aimed to examine the impact of lipids and carbohydrates during the postprandial period and to further elucidate the effect of the repeated biopsy method on inflammatory markers in healthy adipose tissue using the non-caloric placebo. In the present study healthy, young-adults were recruited to consume a beverage containing lipid, carbohydrate or a placebo (water). Adipose tissue gene expression levels in biopsied subcutaneous adipose tissue and circulating levels of adipokines were measured at 2 and 4 h as adipose tissue adaptations to nutrient ingestion have been previously observed during these times ([11], unpublished data [14]). It was hypothesised that lipid ingestion would elicit a higher inflammatory response in adipose tissue than carbohydrate consumption.

## 2. Experimental Section

### 2.1. Participants

Healthy adults aged 18 to 30 years were recruited for the study. Thirty-three male and female adults (24.5 ± 3.3 year (mean ± standard deviation (SD)); body mass index (BMI) 24.1 ± 3.2 kg/m^2^) were randomly assigned to one of three parallel beverage groups, placebo, lipid or carbohydrate. Informed, written consent was obtained from each participant prior to participation in the study, after the nature, purpose and risks of the study as well as their right to withdraw from the study at any time were explained. All experimental procedures were formally approved by the Deakin University Human Research Ethics Committee (2011-027) according to the Declaration of Helsinki. Exclusion criteria included past or present cardiovascular disease, diagnosed diabetes, BMI < 18.5 or >27 kg/m^2^, or hypertension, use of anti-inflammatory medications or supplements (e.g., fish oil).

### 2.2. Experimental Design

Participants were provided with a meal to consume on the evening prior to the day of the trial, in order to standardise nutrient intake, and were instructed to abstain from alcohol, caffeine, tobacco and exercise for 24 h before the test day. On the morning of the trial, participants arrived in a fasted state. Height, weight and waist circumference were measured, then following 30 min of supine resting, an adipose tissue sample was collected from the lateral periumbilical region of the subcutaneous abdominal under local anaesthesia (Xylocaine 1%) by percutaneous needle biopsy technique [15] modified to include suction [16]. Tissue samples were washed in ice cold Phosphate Buffered Saline (PBS) to eliminate blood, then immediately frozen and stored in liquid nitrogen for later analysis. Participants were then fitted with a cannula in the anti-brachial vein of the non-dominant arm and a (0 h) blood sample was taken for serum. All serum samples were stored at −80 °C until analysis for insulin and metabolites including amino acids and fatty acids by mass spectrometry and adipokine analysis via multiplex array.

Participants were randomised to consume either lipid, carbohydrate or placebo beverages (Table S1) as a bolus, in a time period not exceeding 15 min. Subsequent adipose tissue and blood samples were collected at 2 h and 4 h following the meal ingestion. To avoid additional regional inflammation, on each occasion the biopsy needle was angled away 90° from the previous biopsy site.

### 2.3. Beverage Preparation (Table S1)

All beverages contained 35 mg non-caloric sweetener (aspartame) and 185 μL vanilla essence for palatability and to mask macro-nutrient composition. Participants remained blinded to the composition of their beverage throughout the experimental period. The placebo beverage contained the sweetener and flavouring in 350 mL water (0 kJ). The carbohydrate beverage, containing 1856 kJ, was prepared with 116 g maltodextrin (a high glycaemic-index carbohydrate made up of glucose units and is easily metabolised), dissolved in water up to a total volume of 350 mL. The lipid beverage had 1988 kJ and was an emulsion of 143 mL full-fat dairy cream and water prepared to a total volume of 350 mL. Macronutrient composition of the beverages are shown in Supplemental Table S1.

### 2.4. Serum Insulin and Adipokine Analysis

Serum insulin was determined using the Human Insulin Specific RIA kit HI-14K (Merck Millipore, Billerica, MA, USA) according to manufacturer’s instructions. Briefly, serum was incubated overnight with 125I-insulin and human insulin antibody at room temperature. Cold precipitating reagent was added to the sample, vortexed and incubated for 20 m at 4 °C. Tubes were centrifuged at 3000 *g*, and 4 °C for a further 20 m then read on a Cobra II Auto Gamma Counter.

Serum leptin, adiponectin and tumor necrosis factor-α (TNF-α). were measured using the Human Adipokine Kit (Adiponectin HADK1-61K-A, Leptin & TNF-α HADK2-61K-B, Millipore, Billerica, MA, USA) according to manufacturer’s instructions. Briefly 25 µL of each sample was added to wells containing reaction beads and incubated with agitation on a plate shaker overnight at 2–8 °C. The wells were washed; detection antibodies and streptavidin-phycoerythrin were added to each well and incubated with agitation for 30 min at room temperature, then washed again. The beads were resuspended with 100 μL sheath fluid and read on the Bio-Plex array reader (Bio-Rad Laboratories, Sydney, NSW, Australia). All samples were run in duplicate and the coefficient of variation (CV) was calculated; the mean CVs were between 5% and 7%. Participants acted as their own controls (0 h) and adipokine levels are presented as fold change from baseline values.

### 2.5. Metabolomics Analysis

Metabolites were extracted from serum using cold methanol water and freeze-thaw cycles. The internal standard 2,3,3,3-d_4_-alanine (0.3 μmol/sample) was added, samples freeze-dried (BenchTop K manifold freeze dryer, VirTis, SP Scientific, Warminster, PA, USA) and re-suspended in 80% (v/v) cold methanol-water. Metabolite extraction and derivatisation was performed as described by Smart *et al*. [17]. Briefly, the samples were re-suspended in sodium hydroxide solution (1 M (mol/L)) and mixed with methanol and pyridine. Methyl chloroformate (MCF) was added twice and derivatives were separated with chloroform. Sodium bicarbonate solution (50 mM (mmol/L)), was added, the aqueous layer removed and dehydrated with anhydrous sodium sulphate. MCF derivatives were analysed in an Agilent GC7890 system coupled to a MSD5975 mass selective detector (EI) operating at 70 eV. The ZB-1701 gas chromatography (GC) capillary column (30 m × 250 μm id × 0.15 μm with 5 m guard column, Phenomenex, Lane Cove, NSW, Australia).

AMDIS software was used for identifying metabolites using an in-house MCF mass spectra library. The relative abundance of metabolites was determined by ChemStation (Agilent Technologies, Santa Clara, CA, USA) by using the GC base-peak value of a selected reference ion. Values were normalised using an internal standard (2,3,3,3-d_4_-alanine). The data mining and normalisation were automated in R software as described in Smart *et al*. [17] and Aggio *et al*. [18].

### 2.6. RNA Extraction

RNA was extracted using the RNeasy Lipid Tissue Mini Kit (Qiagen Inc., Hilden, Germany) following the manufacturer’s protocol. Briefly, frozen tissue samples (30–100 mg) were homogenised using the Next Advance Bullet Blender tissue homogeniser (Lomb Scientific, Taren Point, NSW, Australia) and 1 mm zirconia/silica beads (Daintree Scientific, St. Helens, Tasmania, Australia) in Qiazol reagent and then incubated for 2–3 min at room temperature with chloroform then centrifuged at 8000 *g* for 15 min at 4 °C. The appropriate RNA phase was collected and ethanol was added prior to washing and elution through the column. On-column DNase treatment (Qiagen) was performed. Total RNA quality and concentration was determined using the Nanodrop ND-1000 (Nanodrop Technologies, DE, USA). The ratio of absorbance at 260 nm and 280 nm was used to assess the purity of the RNA samples. A 260/280 ratio of >1.9 was considered acceptable.

### 2.7. Reverse Transcription and Real-Time-PCR

First strand cDNA was generated from 0.1 µg total RNA using the High Capacity RNA-to-cDNA kit (Applied Biosystems, Foster City, CA, USA). Analysis of gene expression was performed using the CFX384™ Real-Time polymerase chain reaction (PCR) Detection System (Bio Rad Laboratories) using gene specific primers (Supplemental Table S2) designed using Primer Express 3.0 (Applied Biosystems) software. Primer sequence specificity was confirmed using Basic Local Alignment Search Tool (BLAST) and melt curve analysis was performed on each run to confirm the amplification of a single product. Each sample was analysed in duplicate and negative, positive and no template controls were included.

To compensate for variations in input cDNA amounts and efficiency of reverse transcription, results were normalised to human ribosomal 18S mRNA. 18S expression was unaltered across all time-points (data not shown) hence it was considered an appropriate endogenous control to correct for any variation in cDNA concentrations.

### 2.8. Statistical Analysis

Statistical analysis was conducted using SPSS version 17.0 for Windows (SPSS Inc., Chicago, IL, USA). Data is expressed as mean ± SD or standard error of the mean (SEM), as reported. Participant characteristics were compared using a one-way analysis of variance (ANOVA). Data were analysed by repeated measures two-way ANOVA with beverage as the between-subjects factor and time as within-subjects repeated factor. Where interaction was found, post hoc comparisons were performed as *t*-tests with Bonferroni adjustment for multiple comparisons. The number of participants included was estimated to allow an 80% power to detect a difference in postprandial monocyte chemoattractant protein-1 (MCP-1) gene expression levels in adipose tissue between 0 and 4 h as that had been observed in our previous studies (unpublished data, [14]) at a significance level of *p* < 0.05.

## 3. Results

### 3.1. Participants’ Baseline Characteristics

No differences existed between groups with regard to age, height (171.2 ± 12.1 cm), weight (71.0 ± 12.2 kg), BMI, or waist to hip ratio (WHR) (0.85 ± 0.06) (Table 1). Baseline serum levels of the adipokines leptin, adiponectin and TNF-α were not different between groups (Table 1).

**Table 1:** Participants’ baseline characteristics.

	Total	Placebo	Carbohydrate	Lipid	*p-*value
Male	12	3	4	5	****
Female	21	8	7	6	
Age (year)	24.5 ± 3.3	24.8 ± 3.1	23.9 ± 2.9	24.6 ± 3.9	
Height (cm)	171.2 ± 12.1	170.7 ± 10.3	168.3 ± 9.2	174.6 ± 15.6	0.802
Weight (cm)	71.0 ± 12.2	70.4 ± 11.5	67.8 ± 11.5	74.9 ± 12.9	0.476
BMI (kg/m^2^)	24.1 ± 2.7	24.0 ± 1.8	23.8 ± 3.0	24.6 ± 3.2	0.390
Waist (cm)	80.3 ± 7.9	79.7 ± 6.3	79.7 ± 10.2	81.4 ± 7.1	0.802
Hip (cm)	95.0 ± 7.5	95.8 ± 6.3	93.4 ± 7.3	95.6 ± 9.0	0.859
WHR	0.85 ± 0.06	0.83 ± 0.05	0.85 ± 0.08	0.85 ± 0.06	0.715
**Serum Adipokines**					
Leptin (ng/mL)	11.1 ± 10.6	9.8 ± 2.5	12.6 ± 4.5	12.4 ± 4.4	0.854
Adiponectin (µg/mL)	7.1 ± 3.5	8.4 ± 4.0	5.4 ± 2.6	7.6 ± 3.4	0.203
TNF-α (pg/mL)	2.7 ± 1.1	2.3 ± 1.3	3.0 ± 0.9	2.7 ± 1.2	0.514

Data are presented at mean ± SD; BMI = Body mass index; WHR = waist to hip ratio; TNF-α = tumor necrosis factor-α; Participant baseline characteristics were compared using a one-way ANOVA.

### 3.2. Serum Analytes Respond Differently to Beverages Differing in Macronutrient Content

**Figure 1:**
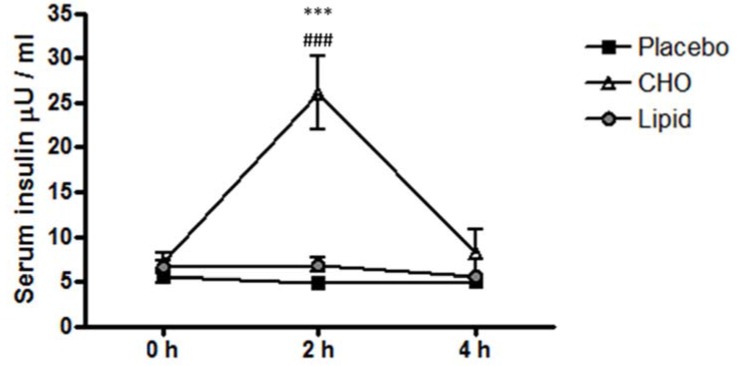
Postprandial response of insulin following consumption of a placebo, carbohydrate or lipid beverage. Levels of insulin were measured in serum at baseline (0 h) and at 2 h and 4 h post ingestion of either a placebo, carbohydrate or lipid beverage. Insulin was determined by Human Insulin Radioimmunoassay (RIA). Data are presented as mean ± standard error of the mean (SEM) (*n* = 11). Interaction; *** *p* < 0.001 *versus* placebo, ^###^ *p* < 0.001 *versus* lipid.

Baseline serum insulin levels did not significantly differ between groups. Postprandial insulin levels increased 3.6-fold in response to carbohydrate ingestion at 2 h compared with baseline (*p* < 0.0001). The placebo and lipid beverages had no impact on circulating insulin concentrations at 2 and 4 h (Figure 1).

**Figure 2:**
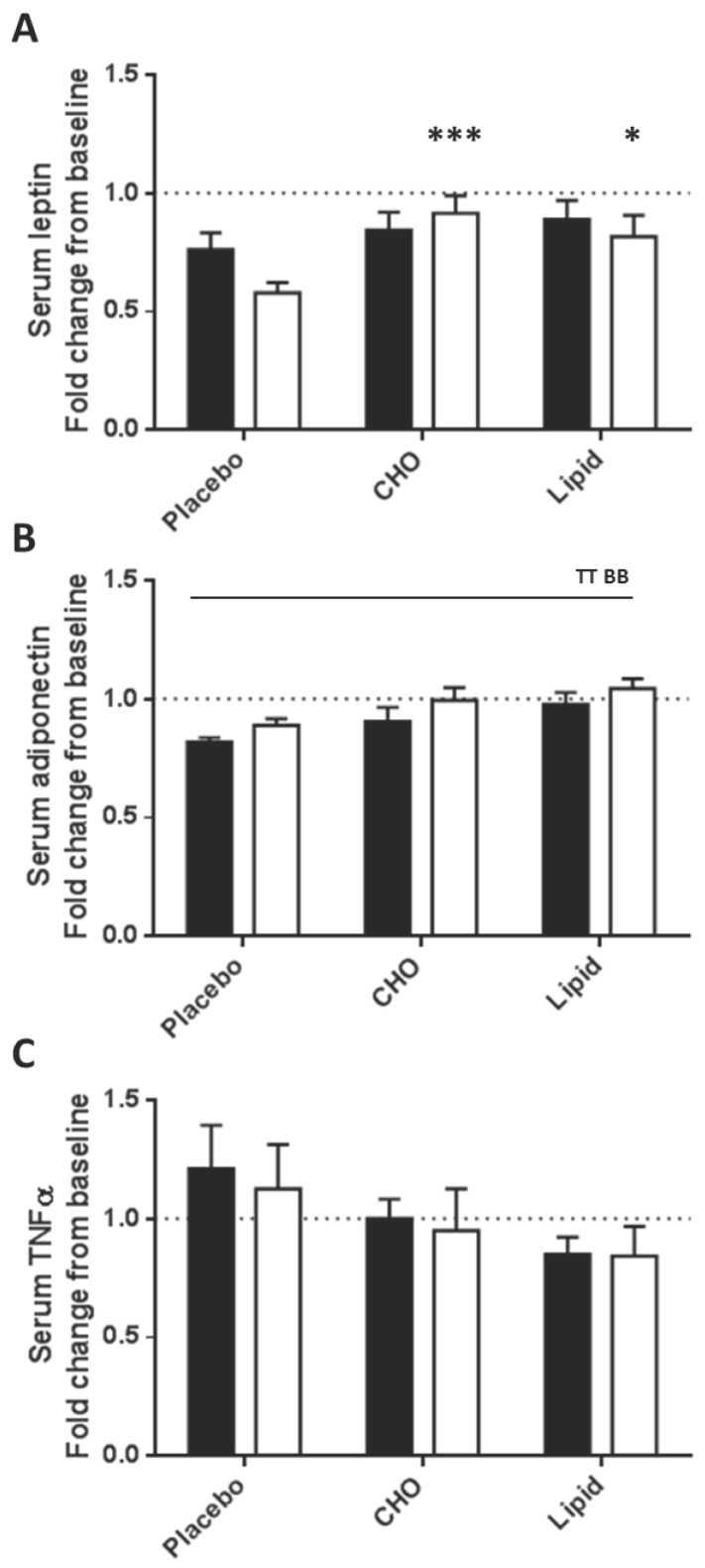
Serum levels of leptin (**A**); adiponectin (**B**) and TNF-α (**C**) were measured at baseline (0 h—set to 1; dotted line) and at 2 h (black bars) and 4 h (open bars) post ingestion of either a placebo, carbohydrate or lipid beverage. Serum adipokine levels were determined using a multiplex array system. Data are presented as mean fold change from baseline (set at 1) ± standard error of the mean (SEM) (*n* = 11). Interaction; * *p* < 0.05; *** *p* < 0.001 *versus* placebo. Main effects across total sample (indicated by black line); TT = time effect *p* < 0.01; BB = beverage effect *p* < 0.01.

Serum leptin levels were lower in the placebo group at 4 h compared with the carbohydrate and lipid groups (0.6 to 0.7-fold, *p* = 0.03; Figure 2A). Main effects showed that serum adiponectin levels decreased at 2 h in all groups (*p* = 0.003), but levels were 15% lower in the placebo group compared with the lipid group (*p* = 0.007; Figure 2B). Serum TNF-α levels were unchanged in the postprandial period for all three beverages (Figure 2C).

**Figure 3:**
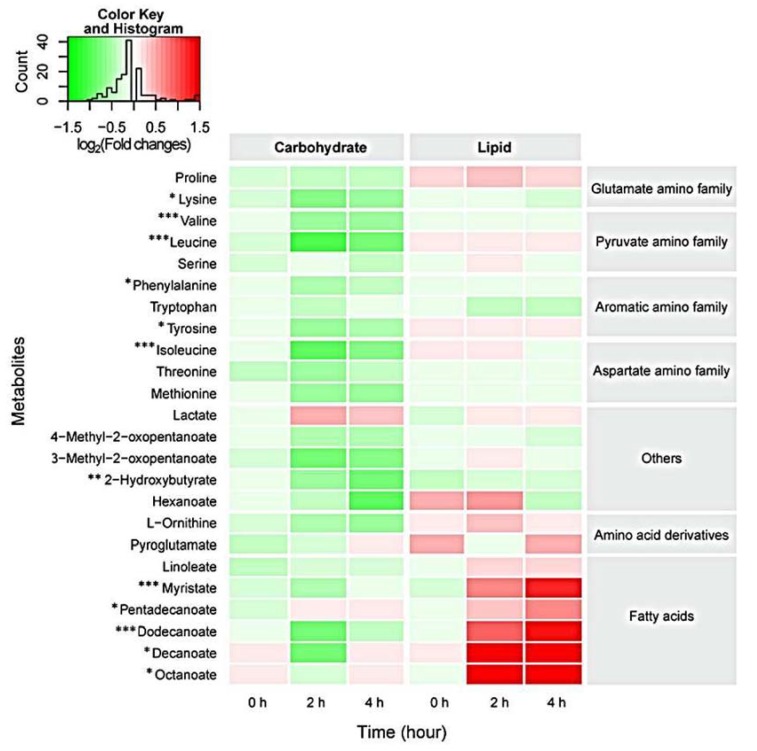
Relative metabolite concentrations extracted from human serum following the consumption of carbohydrate and lipid beverages across 4 h. Metabolite concentrations are given relative to the placebo group, using a log2 scale. Deeper red colours (positive values) indicate that metabolite concentrations were increased in response to different beverage types while green shades (negative values) indicate decreased concentrations. The colour key on the top left is superimposed with a histogram that counts the relative concentrations of all the metabolites. Only the metabolites for which there was a statistically significant change in concentration between beverage types (2-way ANOVA: *p*-value < 0.05) are shown. * Indicate significant interactions between beverage and time, * *p* < 0.05, ** *p* < 0.01, *** *p* < 0.001.

In the hours following ingestion, the availability of metabolites was different for each beverage. Serum free fatty acids levels were increased at 2 and 4 h following the consumption of the (dairy) lipid beverage; myristate (1.7 to 2.1-fold, *p* < 0.0001), dodecanoate (laurate) (2.2 to 2.5-fold, *p* < 0.0001), decanoate (5.9 to 6.1-fold, *p* < 0.05) and octanoate (3.3 to 4.7-fold, *p* < 0.05). These medium to shorter chain fatty acids are typical of dairy fats. No changes were found in the free fatty acid levels measured for the placebo and carbohydrate beverage groups (Figure 3).

Serum amino acid levels (Figure 3) decreased at 2 and 4 h after the carbohydrate beverage compared with the placebo group, indicating insulin-induced amino acid uptake by tissues; lysine (0.6 to 0.7-fold, *p* < 0.05), leucine (0.7 to 0.8-fold, *p* < 0.001), isoleucine (0.8-fold, *p* < 0.0001), phenylalanine (0.7 to 0.8-fold, *p* < 0.05), tyrosine (0.7 to 0.9-fold, *p* < 0.05) and valine (0.7-fold, *p* < 0.0001). Serum amino acid levels remained unchanged in the lipid group. The levels of serum hydroxybutyric acid, a keto-acid and marker of fasting, were increased at 4 h in the placebo (1.7-fold, *p* < 0.001) and lipid groups (1.8-fold, *p* < 0.01), yet were unchanged at 4 h in the carbohydrate group compared with baseline values. Thus, reduced levels of serum hydroxybutyric acid were observed in the carbohydrate group compared with the placebo and lipid groups at 4 h (Figure 3).

### 3.3. Subcutaneous Adipose Tissue Inflammatory Gene Expression Levels Were Equally Increased after Placebo, Carbohydrate and Lipid Beverages

Adipose tissue inflammatory mRNA expression levels were increased in adipose tissue with the placebo, carbohydrate and lipid beverages, with no differences between beverage groups; MCP-1 (27 to 80-fold, *p* < 0.0001), TNF-α (2 to 3.7-fold, *p* < 0.0001) and IL-6 (83 to 360-fold, *p* < 0.0001) (Table 2). Gene expression levels of cluster of differentiation 68 (CD68) were decreased by at least 0.73-fold at 2 h in all groups compared with baseline (*p* = 0.042) (Table 2). Visfatin levels increased over time in all beverage groups (*p* < 0.0001) (Table 2).

**Table 2:** Adipose tissue reverse transcription polymerase chain reaction (RT-PCR)—Fold change from baseline.

		Placebo	Carbohydrate	Lipid	P1	P2	P3
**Adipokines**							
Leptin	2 h	0.69 ± 0.09	1.01 ± 0.12	1.03 ± 0.13	0.129	**0.001**	**0.027**
	4 h	0.57 ± 0.07	0.85 ± 0.10	0.86 ± 0.11			
Adiponectin	2 h	0.86 ± 0.08	0.69 ± 0.08	0.88 ± 0.17	0.658	**0.007**	0.389
	4 h	0.84 ± 0.09	0.67 ± 0.07	0.90 ± 0.20			
Resistin	2 h	1.07 ± 0.08	1.39 ± 0.20	1.17 ± 0.15	0.716	0.079	0.328
	4 h	0.90 ± 0.08	1.13 ± 0.21	1.04 ± 0.18			
Chemerin	2 h	0.90 ± 0.07	1.09 ± 0.08	0.89 ± 0.09	0.424	0.105	0.291
	4 h	0.80 ± 0.06	0.92 ± 0.10	0.91 ± 0.12			
Visfatin	2 h	3.59 ± 0.73	4.19 ± 1.10	4.74 ± 1.04	0.803	**<0.0001**	0.718
	4 h	6.51 ± 1.29	5.41 ± 1.43	7.10 ± 1.45			
PAI-1	2 h	1.16 ± 0.15	1.08 ± 0.14	1.09 ± 0.20	0.830	0.064	0.978
	4 h	0.83 ± 0.11	0.96 ± 0.11	0.97 ± 0.13			
**Inflammatory Molecules**							
MCP1	2 h	27.1 ± 6.6	34.7 ± 11.8	47.3 ± 12.7	0.886	**<0.0001**	0.832
	4 h	80.8 ± 20.8	69.0 ± 21.6	78.8 ± 22.6			
TNF-α	2 h	3.65 ± 1.04	3.31 ± 0.74	2.89 ± 0.47	0.565	**<0.0001**	0.701
	4 h	2.05 ± 0.46	3.14 ± 0.54	2.35 ± 0.37			
IL-6	2 h	83.3 ± 28.9	241.9 ± 106.4	258.0 ± 101.3	0.414	**<0.0001**	0.602
	4 h	259.7 ± 65.3	359.7 ± 138.4	241.7 ± 80.5			
CD68	2 h	0.87 ± 0.15	0.73 ± 0.10	0.73 ± 0.10	0.243	**0.042**	0.280
	4 h	1.06 ± 0.19	0.98 ± 0.18	0.63 ± 0.12			

Values are mean ± standard error of the mean (SEM) fold change mRNA expression at 2 h and 4 h compared with baseline (0 h). Expression is relative to ribosomal 18S. Repeated measures ANOVA was performed with beverage as a between subjects factor and time as the repeated factor. P1 = time × beverage interaction; P2 = main time effect; P3 = main beverage effect; PAI-1 = plasminogen activator inhibitor-1, MCP-1 = monocyte chemoattractant protein-1, TNF-α = tumor necrosis factor-α, IL-6 = interleukin-6, CD68 = cluster of differentiation 68.

### 3.4. Subcutaneous Adipose Tissue Adipokine Gene Expression Levels Differed in Response to Energy/Nutrient-Containing Beverages Compared with the Placebo

Main effects were observed for time and beverage for adipose tissue leptin mRNA expression (Table 2). Main effects showed that leptin levels decreased over time, but the beverage effect demonstrated levels were lower in the placebo group. While a statistically significant interaction effect was not observed, the placebo group appeared to be the main contributor to the reduced expression of leptin observed in the main effects and the trends were similar to the effects seen in serum leptin levels (Table 2). Adiponectin mRNA expression levels decreased 0.8-fold in adipose tissue at 2 h (*p* = 0.026) and 4 h (*p* = 0.045) compared with baseline in all beverage groups (Table 2). There were no changes in adipose tissue resistin, chemerin or plasminogen activator inhibitor-1 (PAI-1) gene expression levels.

## 4. Discussion

Single meals differing in macronutrient composition are likely to generate a tailored transcriptional and translational response within adipose tissue. This enables the adipocyte to process and regulate the storage of the available macronutrients, whilst adapting the expression of secreted adipokines that are necessary for local and systemic regulation of metabolism and immune function [3,4,5,6]. Despite the importance of these fundamental processes as regulators of adipose tissue triacylglycerol storage [1,19] and chronic disease risk [9,10], few studies have addressed how adipose tissue inflammation are regulated by a single meal. In the present study, healthy volunteers were randomised to consume one of three beverages, placebo (water), carbohydrate or lipid. The subsequent impact on adipose tissue gene expression levels was measured in serial needle aspirated biopsy samples harvested from abdominal subcutaneous adipose tissue at 2- and 4-h post meal ingestion.

Increased inflammatory gene expression levels in adipose tissue were found, without an increase in circulating TNF-α levels in each beverage group. Circulating TNF-α levels increased in healthy, obese and diabetic individuals following a high-fat meal, but not following a high carbohydrate meal [20,21,22]. In contrast, the present study observed no change after 4 h in serum TNF-α level in any of the beverage groups, despite increased adipose tissue TNF-α mRNA levels. However the population sample in the current study was consistent with another study in young healthy adults; where postprandial TNF-α was unchanged [23]. The discordance between adipose tissue and serum TNF-α levels suggests that this might be due to a biopsy-induced, local inflammation in the adipose tissue, rather than a postprandial effect. However, further studies that investigate repeated biopsies from multiple incision sites are required to assess whether this result is a true impact of the biopsy effect or a lack of translation from RNA expression levels to a secreted protein response.

Meneses *et al*. [11] reported increased adipose tissue inflammatory gene expression levels (including MCP-1, IL-6, interleukin-1β (IL-1β)) in adipose tissue 4 h following both a high fat and a low-fat, high carbohydrate meal, in individuals with metabolic syndrome (MetS) using repeated biopsies. However, the actual sites from which the repeat biopsies were taken from were not reported. Additionally, Pietraszek *et al*. [12] also detected inflammatory changes following consumption of meals combined with either coconut oil (rich in saturated fatty acids, (SFA)) or macadamia oil (rich in monounsaturated fatty acids (MUFA)) in adipose tissue of middle aged adults, who were either relatives of people with type 2 diabetes (median years (95% confidence interval (CI)); 55.0 (48.0–62.0)) or age-matched controls (46.0 (37.5–57.5)). However, no placebo or non-nutrient treatment was utilised to test the effect of the procedure. Similar to the present study, the biopsies were reported to be taken through a 1 cm incision with 5 cm between pre and post meal biopsy sites. However, a recent study by Kruse *et al*. [24] showed increased inflammatory gene expression levels in adipose tissue at 4 h following a meal rich in rapeseed oil but not olive oil in moderately obese men aged 39 to 63 years. Biopsy sites and techniques were not reported. The biopsies in the current study were taken from the same incision, but each at a 90° angle from the previous biopsy, and approximately 4–5 cm from the incision site (see Figure S1). A recent study in skeletal muscle, with a similar biopsy protocol to the present study, showed increased gene expression levels of inflammatory markers IL-6 and IL-6 receptor (IL-6R), with the repeated biopsies from the same incision site [13]. Therefore, while precautions were taken to reduce the influence of biopsy-induced local inflammation, the biopsy technique used in the current study may not have been adequate for differentiating between biopsy-induced inflammation and possible acute inflammatory responses to nutrients in adipose tissue.

Contrary to the findings with the acute inflammatory markers, levels of the adipokine leptin were differentially impacted by the nutrient beverages compared with the placebo. Adipose tissue leptin mRNA expression levels were reduced in the placebo group, compared with both nutrient beverage groups, which was consistent with the effect observed in serum. Circulating leptin levels were 42% lower at 4 h compared with baseline in the placebo beverage group. Human serum leptin levels decrease during fasting in both normal weight and obese individuals [7,8,25]. Adipose tissue adiponectin mRNA expression remained unchanged after all beverages, which is consistent with other findings in healthy and non-diabetic obese people [10]. However, main time and beverage effects were observed for serum adiponectin. Protein levels of adiponectin were lowered over time, and were particularly influenced by the placebo group when compared with the lipid group; an effect that has also been recently demonstrated in middle-aged males with metabolic syndrome after a cream-based oral fat-load [26].

Expression levels of metabolic genes previously identified to be altered in adipose tissue during the postprandial period (unpublished data, [14]) were also measured to test whether the biopsy effect was consistent for all adipose tissue responses. Levels of the metabolic genes were decreased in adipose tissue following the carbohydrate and lipid beverages and were unchanged in the placebo group; further indicating that some adipose tissue responses were unaffected by the biopsy procedure (insulin receptor substrate (IRS2), pyruvate dehydrogenase kinase, isozyme 4 (PDK4) and phosphoinositide-3-kinase interacting protein 1 (PIK3IP1) (data not shown)).

Availability of nutrients and insulin levels are important determinants of a postprandial response. While metabolomic parameters can be variable between healthy young adults [27], the present study observed increased serum insulin levels in the carbohydrate group that were associated with serum amino acid clearance, indicating uptake of nutrients by the tissues in the postprandial period. Whereas, the lipid beverage group exhibited increased serum fatty acids in the postprandial period, but not insulin-induced clearance of amino acids. The placebo beverage increased serum 2-hydroxybutyric acid, a metabolite of ketosis and an indicator of fasting [28]. Despite the obvious differences in the available metabolites after each of the nutrient beverages, no inflammatory changes were observed beyond those that were induced by the biopsy as detected in the water beverage group.

Investigating the response of adipose tissue to different nutrients should give insight to how it regulates metabolic homeostasis but due consideration needs to be given to methodology. One limitation is that it is not possible to sample different/many different adipose tissue sites in human studies and in the case of the current study, we have highlighted a technical matter of repeated biopsy from the same site. The increased levels of inflammatory genes in adipose tissue measured in all beverage groups, suggest that the repeated biopsy method induced this effect, rather than being induced by nutrient ingestion.

## 5. Conclusions

It was hypothesised that the lipid meal would result in heightened inflammation compared with carbohydrate or placebo. However, the inflammation elicited by the biopsy method is likely to have interfered with the identification of whether an inflammatory response exists in adipose tissue as a consequence of nutrient ingestion. It is recommended for all future studies of this nature to report biopsy sites and techniques in order to differentiate biopsy-induced inflammation from nutrient-induced inflammation. Despite this apparent technical limitation, the present study showed differences in adipose tissue gene expression levels that appeared to be unaffected by the biopsy, indicating that adipose tissue does acutely respond to nutrient ingestion, and this response is somewhat differential and dependent on insulin levels and metabolite availability. The findings of this study warrants further investigation into the acute metabolic adaptations of adipose tissue and of the comparison of these results with responses in metabolically compromised individuals.

## Supplementary Files

Supplementary File 1
