# Supplementary material for: Postprandial Responses to Lipid and Carbohydrate Ingestion in Repeated Subcutaneous Adipose Tissue Biopsies in Healthy Adults
Source: Nutrients. 2015 Jul 1;7(7):5347–61. doi: 10.3390/nu7075224 (PMC4517001; doi:10.3390/nu7075224)
Supplement: Supplementary File 1 [file nutrients-07-05224-s001.docx]

**Supplementary Materials**

**Table S1.** Nutrient composition of the beverages.

|  | **TE (kJ)** | **TE from Lipid (kJ)** | **TE from Carbohydrate (kJ)** | **TE from Protein (kJ)** | **TE (Cal)** | **TE from Lipid (Cal)** | **TE from Carbohydrate (Cal)** | **TE from Protein (Cal)** | **% TE Fat** | **% TE  Carbohydrate** | **% TE  Protein** |
| --- | --- | --- | --- | --- | --- | --- | --- | --- | --- | --- | --- |
| **Placebo** | 0 | 0 | 0 | 0 | 0.0 | 0.0 | 0.0 | 0.0 | 0 | 0 | 0 |
| **Carbohydrate** | 1856 | 0 | 1856 | 0 | 442 | 0.0 | 442 | 0.0 | 0 | 100 | 0 |
| **Cream (lipid)** | 1988 | 1852 | 68.6 | 68.6 | 473 | 441 | 16 | 16 | 93 | 3.5 | 3.5 |

kJ = kilojoule, Cal = Calories, TE = Total Energy.

**Table S2.** Human primer sequences.

| **Gene** | **Accession No.** | **Forward** | **Reverse** |
| --- | --- | --- | --- |
| *18S* | NR_003286.2 | TTCGGACGTCTGCCCTATCAA | ATGGTAGGCACGGCGACTA |
| *Adiponectin* | NM_004797 | GGGCATCCGGGCCATA | TTTCACCGATGTCTCCCTTAGG |
| *CD68* | NM_001251.2 | ATCCCCACCTGCTTCTCTCA | GAGGTCCTGCATGAATCCAAA |
| *Chemerin* | NM_002889 | CGCCCCGCGAGAAGAAGAGC | CACCCTGGCCGTCCTTCCCT |
| *Leptin* | NM_000230 | CACCAAAACCCTCATCAAGACAA | GACTTTCTGTTTGGAGGAGACTGACT |
| *MCP1 (CCL2)* | NM_002982.3 | CGCCTCCAGCATGAAAGTCT | GGAATGAAGGTGGCTGCTATG |
| *PAI-1* | NM_000602 | GTGGGAAGGCCAGTGTCCACCCA | CATCCTTGGGCACGGGAGAGCT |
| *Resistin* | NM_001193374 | CCTTACAGGGAAGACCACCACCCTC | CGCCCATCTTCCAGTGCTTGTCTT |
| *TNF-α* | NM_000594 | GGAGAAGGGTGACCGACTCA | TGCCCAGACTCGGCAAAG |
| *Visfatin* | NM_005746 | GGTCTGGAATACAAGTTACATGATTTTG | TTGAAGTTAACCAAGTGAGCAGATG |

Gene specific primers designed using Primer Express 3.0 (Applied Biosystems) software. Primer sequence specificity was also confirmed using Basic Local Alignment Search Tool (Blast).


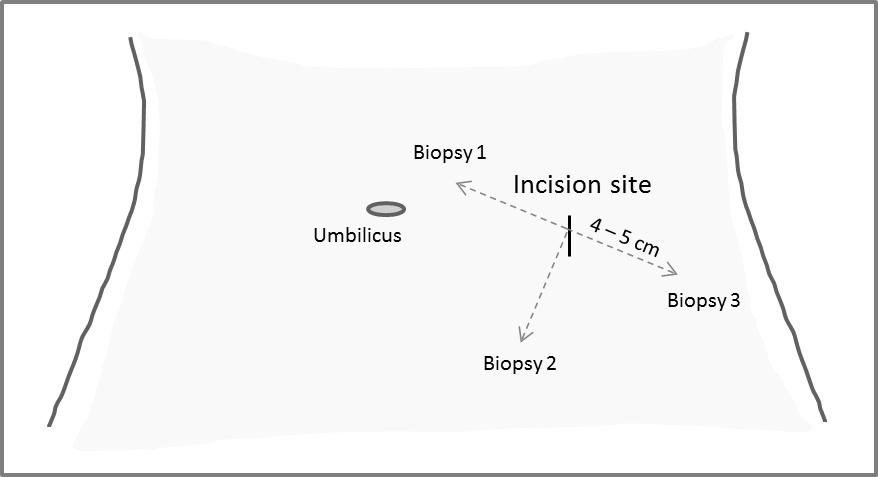


**Figure S1.** Biopsy site technique used for the collection of adipose tissue samples.

© 2015 by the authors; licensee MDPI, Basel, Switzerland. This article is an open access article distributed under the terms and conditions of the Creative Commons Attribution license (http://creativecommons.org/licenses/by/4.0/).
